# Transcriptomic changes underlying glucocorticoid-induced suppression of milk production by dairy cows

**DOI:** 10.3389/fgene.2022.1072853

**Published:** 2022-12-06

**Authors:** Anna Sadovnikova, Sergio C. Garcia, Josephine F. Trott, Alice T. Mathews, Monica T. Britton, Blythe P. Durbin-Johnson, Russell C. Hovey

**Affiliations:** ^1^ Department of Animal Science, University of California, Davis, Davis, CA, United States; ^2^ School of Medicine, University of California, Davis, Sacramento, CA, United States; ^3^ School of Life and Environmental Sciences, University of Sydney, Sydney, NSW, Australia; ^4^ UC Davis Bioinformatics Core, University of California, Davis, Davis, CA, United States

**Keywords:** dexamethasone, lactose, alpha-lactalbumin, lactation, inflammation

## Abstract

Milk production by dairy cows is sensitive to increased levels of stress hormones such as glucocorticoids (GC) that also regulate the transcription of several genes required for milk synthesis. Whereas previous studies identified that an exogenous GC such as dexamethasone (DEX) transiently suppresses milk yield in several species without any pronounced effect on milk protein or fat percentage, the mechanism underlying this effect has not been established. In this study we sought to establish changes within the mammary glands of non-pregnant dairy cows in their second lactation (*n* = 3–4; 648–838 kg) following a single dose of exogenous DEX. Changes in the udder were monitored by serial biopsy of alternating quarters, concurrent with quarter-level monitoring of milk yield and composition. Dexamethasone increased serum glucose levels from 12–36 h (*p* <0 .05), reduced milk yield from 12–48 h (*p* <0 .05), increased % milk protein content at 24 h post-DEX, and transiently decreased both milk lactose and α-lactalbumin content, while not altering the level of milk fat. After 72 h, all aspects of milk production had returned to pre-treatment levels. Transcriptomic changes in the mammary glands in response to DEX were identified by RNA sequencing followed by differential gene expression analysis. Coincident with the milk yield and composition changes was the differential expression of 519 and 320 genes at 12 and 24 h after DEX (adjusted *p* <0 .05), respectively, with the return of all gene expression to baseline levels by 72 h. Among the transcriptomic changes in response to DEX, there was notable downregulation of elements in the lactose synthesis pathway, specifically *AQP3, GALE* and *LALBA* (α-lactalbumin) at 12 h, and sustained downregulation of *LALBA* at 24 h. One gene in the pathway, *UGP2*, was upregulated at 12–24 h post-DEX. This work supports the hypothesis that there is a direct relationship between the response to DEX and the concurrent suppression of milk yield due to the reduced synthesis of α-lactalbumin and lactose by the mammary epithelium. The ability of glucocorticoids to modulate the homeorrhetic requirements for glucose during stressful states concurrent with immune activation bears significance for dairy animals as well as a broad range of lactating mammals.

## Introduction

Stress can suppress milk production by dairy animals (Romero et al., 2015; Hong et al., 2019) in association with a range of negative outcomes, including depressed feed intake and increased susceptibility to mastitis and metritis (Menta et al., 2022). The overarching stress response is mediated, in large part, by the endocrine environment including reduced responsiveness to oxytocin (Bruckmaier and Wellnitz, 2008), immunosuppression (Waller, 2000), glucose sparing, and gluconeogenesis (Sapolsky et al., 2000). Many of these changes are coordinated by increased circulating glucocorticoids (GC) that are elevated in response to stressors, including change of environment, heat stress, transport and disease (Johnson and Vanjonack, 1976).

During lactation the extreme demand for glucose by the mammary glands is part of a homeorhetic/homeostatic balance, that is, coordinated through mechanisms including elevated GC. While GC are essential for the transcription of milk protein genes by mammary epithelial cells (Casey and Plaut, 2007), the extent to which the lactating mammary glands respond to elevated GC remains unclear. Several studies have demonstrated that an acute, high dose of exogenous GC, including a synthetic GC such as dexamethasone (DEX), leads to the abrupt and transient suppression of milk production (Hartmann and Kronfeld, 1973; Shamay et al., 2000b; Babwah et al., 2013), which is more pronounced in cows than goats (Shamay et al., 2000a). Coincident with this DEX-induced suppression of milk yield was a reduction in the extraction of glucose from the circulation by the mammary glands, as determined from arterio-venous difference (Hartmann and Kronfeld, 1973). Further to these findings, Shamay et al. (2000b) identified that the reduction in milk production following DEX was associated with a specific reduction in the proportion of lactose in milk, whereas the level of protein and fat in milk was unchanged. While GC have also been implicated in the regulation of tight junction integrity (Stelwagen et al., 1998), exogenous DEX did not affect the ratio of Na/K in the milk (Shamay et al., 2000b), suggesting that the effect of DEX was not due to altered integrity of these intercellular junctions.

The pronounced and transient effects of a GC such as DEX on the synthesis and composition of milk raise questions about the mechanism(s) underlying this response, including whether it occurs through a systemic mode of action, or through local effects on the mammary glands. To this end, we sought to establish the temporal transcriptomic response within the udder of high-producing dairy cows following an acute exposure to DEX. Our data establish that a primary target of acute DEX exposure is the lactose synthesis pathway, including through the marked down-regulation of α-lactalbumin (LALBA) gene transcription.

## Materials and methods

### Animals and study design

All animal experimentation was approved by the UC Davis Institutional Animal Care and Use Committee. Four non-pregnant Holstein cows were enroled in the study (average 738.2 kg, range 648–838 kg) in their second lactation (average 55 DIM, range 40–64 DIM). None had a prior history of clinical mastitis. Cows were housed in separate pens and were bedded on rice hulls with *ad libitum* access to water and feed. Cows were fitted with rumination collars (SCR Engineers Limited, Israel).

The study period included an 8 days acclimation prior to the single administration of DEX on day 9. Four days prior to DEX, each cow was fitted with an indwelling jugular catheter that was flushed daily with saline and locked with heparinized saline (250 IU per ml). On day 9, each cow was administered a single injection of DEX (40 mg, IM, VetOne, Boise, Idaho) between 19:00 and 21:30, immediately after the first biopsy and the subsequent milking. Blood was collected into vacutainers containing potassium oxalate and sodium fluoride every 12 h out to 5 days post-DEX and was processed by centrifugation at x 2,000 g for 10 min to yield serum that was stored at -80°C.

### Feed intake, composition, and rumination

The lactating cow ration consisted of (w/w, as fed) rolled corn (40.4%), alfalfa hay (32.3%), chopped wheat hay (9.3%), cottonseed (7.7%), almond hulls (7.7%), mineral mix (1.2%), EnerGII supplement (Virtus, 1%), Strata (Virtus, 0.3%), and salt (0.2%). Each cow was offered 40 kg (as fed) of total mixed ration daily, which was delivered as 10 kg portions at 06:00, 12:00, 18:00 and 0:00. Refusals were collected and weighed daily at 18:00 for 5 days prior to, and 4 days following, administration of DEX. Proximate analysis of the ration was performed by a commercial laboratory (DairyOne, Ithaca, NY; Supplementary Table S1).

### Milk collection procedure and milk yield and composition analysis

Cows were milked twice daily, at 12 h intervals (06:00–08:00 and 18:00–20:00) using a portable milking machine that allowed for separate collection of milk from each quarter (QTR). The left rear QTR was designated as QTR1, the left front was QTR2, the left right was QTR3, and the right rear was QTR4. During the experimental period, fore- and hindmilk were collected, weighed, and sampled separately prior to, and following, administration of oxytocin (30U, IV, VetOne, Boise, Idaho), respectively. The fore- and hindmilk from each QTR was then combined and sampled in duplicate. When specified, hindmilk samples were from QTR4. When a biopsy was performed, milking and sampling of all QTR was performed immediately thereafter. After biopsy, some samples contained contaminating blood and were not analyzed for composition, namely QTR1 (0 h post DEX), QTR2 (12 h post DEX), QTR3 (24 h post DEX), and QTR4 (72 h post DEX). Duplicate milk samples were chilled on ice and supplemented with bronopol preservative (Microtabs II, Nelson- Jameson) then stored at 4°C or –20°C. Refrigerated samples were analyzed for lactose, fat, casein, total protein, solids, and somatic cell count (SCC) by a commercial laboratory (DairyOne, Ithaca, NY). Minerals (Na, K, Mg, Ca, Cl, and P) were analyzed in frozen milk samples (DairyOne, Ithaca, NY).

### Milk α-lactalbumin

The content of LALBA in milk sampled at –24, –12, 0, 12, 24, 36, 48, 60, 73, and 84 h relative to DEX was determined using a bovine LALBA ELISA (Bethyl Laboratories, Montgomery, TX United States) per the manufacturer’s instructions. Concentrations were established from a standard curve generated with the provided bovine LALBA, where the resultant absorbance was measured at 280 nm using a Synergy HT microplate spectrophotometer (BioTek, Winooski, VT). All samples were assayed in triplicate.

### Serum glucose

Glucose levels were quantified using a glucose colorimetric assay kit (Cayman Chemical, Ann Arbor, MI) according to the manufacturer’s instructions. The standard curve was prepared with the provided glucose that was serially-diluted. Absorbance was measured at 520 nm using a Synergy HT microplate spectrophotometer (BioTek, Winooski, VT), where all samples were assayed in triplicate.

### Mammary biopsy

One or two cores of tissue were collected using a needle biopsy tool (16 ga. Magnum, Bard, Covington, GA) that was inserted through a small incision in the skin following local anesthesia (0.125% bupivacaine, SC). Sequential biopsy across the experimental period was performed on alternating udder QTR at either time 0 (QTR1), 12 h (QTR2), 24 h (QTR3), or 72 h (QTR4) post-DEX to capture the anticipated full range of the milk yield response (Shamay et al., 2000b). Tissue cores were flash frozen in liquid nitrogen and stored at –80°C. Cows received prophylactic ampicillin (Polyflex, Boehringer Ingelheim, IM) for 3 days spanning the biopsy period.

### RNA isolation, cDNA library preparation and sequencing

Total RNA was isolated from biopsy cores (∼10–50 mg tissue) from 4 cows at 0, 12, and 24 h, and from 3 cows at 72 h, using TRIzol (Invitrogen, ThermoFisher, Waltham, MA) according to the manufacturer’s instructions. The integrity of the total RNA and its yield were confirmed by formaldehyde gel electrophoresis with staining (SybrSafe, Invitrogen) and UV visualization, and spectrophotometry (Nanodrop, ThermoScientific), respectively. Total RNA (5 μg) was treated with DNaseI (Zymo Research, Irvine, CA) and analyzed for quality (Experion RNA StdSens, BioRad, Hercules, CA), where all samples had an RNA integrity value greater than 8.3.

Gene expression profiling was performed using 3′Tag-RNA-Seq. Barcoded sequencing libraries were prepared using the QuantSeq FWD kit (Lexogen, Vienna, Austria) for multiplexed sequencing according to the manufacturer’s recommendations, using 700 ng input RNA and 13 cycles of PCR for final library amplification. Fragment size distribution of the libraries was verified *via* microcapillary gel electrophoresis on a Bioanalyzer 2100 (Agilent, Santa Clara, CA). The library masses were quantified on a Qubit fluorometer (LifeTechnologies, Carlsbad, CA), and pooled in equimolar ratios. The final pool was treated with Exonuclease VII followed by bead clean-up to remove free primer. The pool was quantified by qPCR with a Kapa Library Quant kit (Kapa Biosystems, loaction). Fifteen libraries were sequenced per lane on a HiSeq 4000 sequencer (Illumina, San Diego, CA) with single-end 90 bp reads generating an average of 6 million reads per sample.

### Bioinformatic analyses

Raw reads were processed with HTStream (https://ibest.github.io/HTStream/) to remove adapter and low-quality sequences. On average, 0.2% of reads were removed. The trimmed reads were aligned to the *Bos taurus* UMD3.1 genome with Ensembl gene annotation release 93 using the aligner STAR v. 2.6.0c (Dobin et al., 2013) to generate raw counts per gene. On average, over 97% of the reads aligned to the *B. taurus* genome, and 76% of the trimmed reads uniquely aligned to a *B. taurus* gene. The RNA-seq data was submitted to GEO under the accession number GSE217369.

Prior to analysis, genes having an expression level across all samples of less than 4 counts per million reads were filtered out, leaving 10,241 genes. Differential expression analysis was conducted using the limma-voom Bioconductor pipeline (limma version 3.38.3, edgeR version 3.24.3, R version 3.5.1). The model used within limma was a single-factor ANOVA model for comparisons between timepoints, and a linear regression model for correlations between continuous milk characteristics and gene expression. In all limma analyses, standard errors and estimates of log fold changes were adjusted for within-cow correlations. Gene ontology (GO) enrichment analyses were conducted by Kolmogorov-Smirnov testing as implemented in the Bioconductor package topGO (version 2.32.0.). Gene enrichment analyses were also conducted with ShinyGO 0.76.3 (http://bioinformatics.sdstate.edu/go/), using a background list containing 9,745 of our 10,241 expressed genes that were annotated with a gene symbol. Linear mixed effects models were used to evaluate the correlation between module eigengenes and the phenotype variables of total milk yield, total lactose %, total casein %, total protein %, total solids %, or total fat %.

Genes that were differentially-expressed at 12 and 24 h relative to time 0 (adjusted *p* <0 .05) were filtered by up- or down-log-fold change, then uploaded to the Database for Annotation, Visualization and Integrated Discovery (DAVID v6.8) using the *B. taurus* background list (Huang et al., 2009b; a). After selecting GOTERM_MF_DIRECT, the functional annotation chart was used where a threshold of two genes, an EASE score of 1, fold-enrichment, and false discovery rate (FDR) were selected. Enrichment terms with FDR>0.05 were removed. Genes that were differentially-expressed at 12 and 24 h post-DEX were also aligned with gene lists that were generated for the lactose synthesis pathway, or for the GO terms “tight junctions”, “inflammation”, “response to corticosteroids”, and “regulation of blood vessel diameter” (Ashburner et al., 2000; Lemay et al., 2013; Sadovnikova et al., 2021a; Gene Ontology Consortium, 2021). Upstream regulators of genes that were differentially-expressed at 12 and/or 24 h (adjusted *p* <0 .05) were predicted using Enrichr (https://maayanlab.cloud/Enrichr/) and its Drug Signatures Database.

### Statistical analyses

All data were analyzed as a mixed-effects model with repeated measures using Prism 9 (GraphPad Software, San Diego, CA). Data were checked for normality and homogeneity of variance and transformed where necessary. Cow was the experimental unit, with time (post-DEX) and milk type (fore-vs. hindmilk) being fixed effects. A post-hoc Tukey test was performed for all data except the RNA-seq data where a post-hoc Dunnet test was performed. Significance was declared at *p* <0.05.

## Results

### Effect of DEX on rumination, feed intake, and plasma glucose levels

**FIGURE 1:**
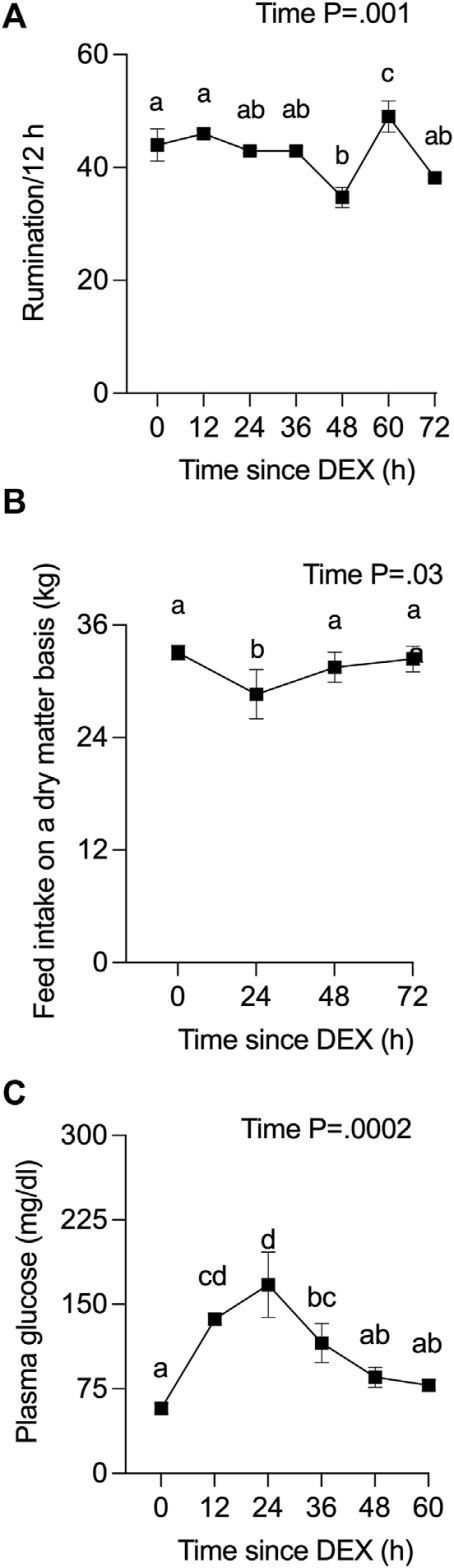
Effect of a single administration of dexamethasone (DEX) on **(A)** rumination, **(B)** daily feed intake, and **(C)** plasma glucose. Time zero represents the average of 12 data points from −24 to 0 h (h) for rumination, the average of −24 and 0 h for feed intake, and the average of −24, −12, and 0 h for plasma glucose. Data are means ± SEM (*n* = 3 cows). a, b, c, d Means with different superscripts are different (*p* <0 .05). The *p*-value for the main effect of time is indicated on each panel when *p* <0.05.

Rumination frequency was captured for cows from 42 h before, to 100 h after, DEX. One cow ceased ruminating by 24 h post-DEX, developed hematochezia starting at 36 h post-DEX, and was removed from the study at that time. All data for that cow are presented in Supplemental Data given that many physiological parameters up to 24 h post-DEX were notably similar to the responses recorded for other cows. The rate of rumination for the three remaining cows varied slightly over the experimental period following DEX (Figure 1A), where there was a small transient reduction in dry matter-adjusted feed intake (Figure 1B) during the 24 h after DEX that then returned to baseline. Plasma glucose levels (Figure 1C) were increased more than 2-fold by 12 h post-DEX (*p* <0 .0001), reaching a peak of 167 mg/dl at 24 h, before they returned to euglycemic values by 48 h after DEX.

### Effect of DEX on milk yield and composition

**FIGURE 2:**
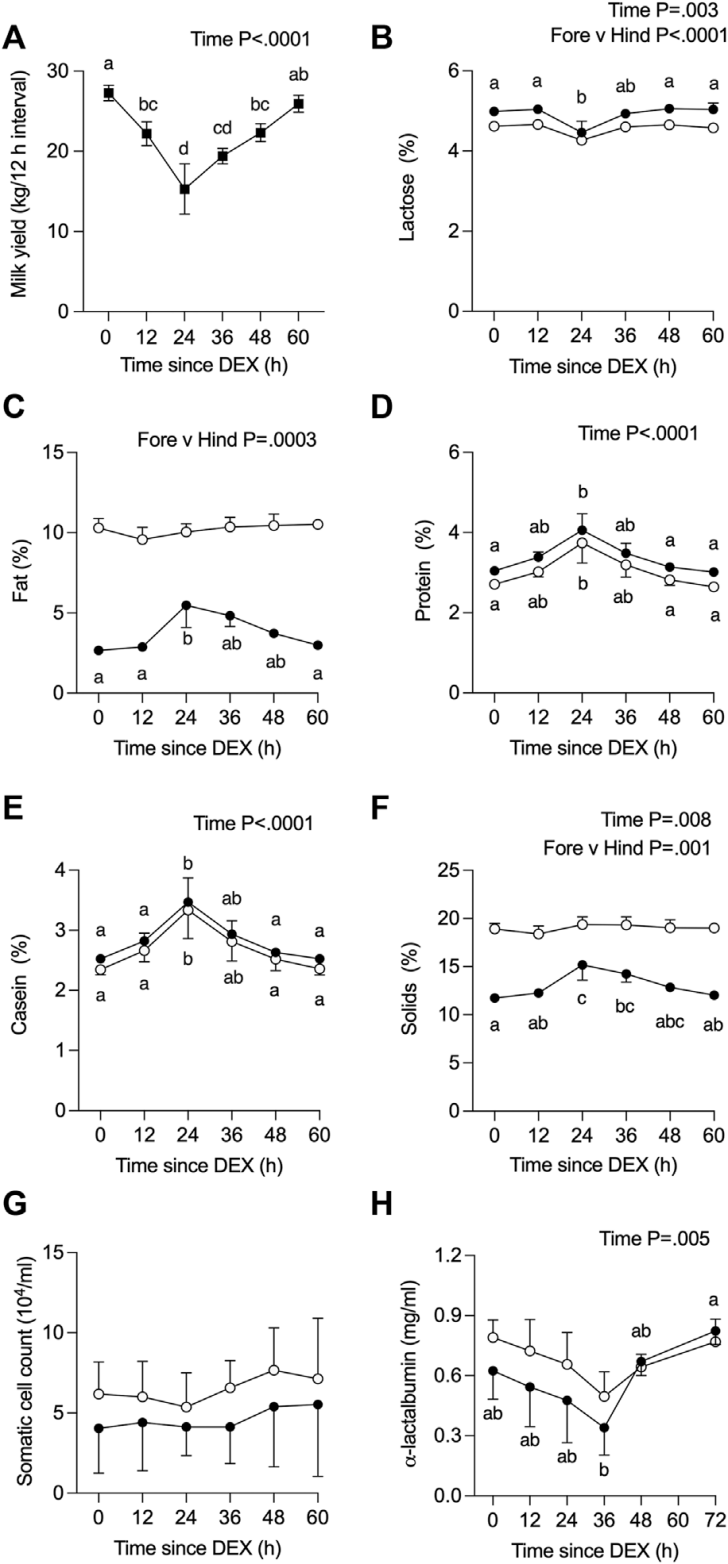
Effect of dexamethasone (DEX) on **(A)** total milk yield, or on components (%) in foremilk (black circles) and hindmilk (open circles) for **(B)** lactose, **(C)** fat, **(D)** protein, **(E)** casein, and **(F)** total solids, as well as **(G)** somatic cell count, and **(H)** α-lactalbumin (LALBA) concentration. Data are means ± SEM (*n* = 3 cows). Time zero represents an average of −36, −24, −12, and 0 h (h) relative to DEX for all components except for LALBA, where the baseline value is the average of −24, −12, and 0 h a, b, c, d Means with different superscripts are different (*p* <0.05). The *p*-value for a main effect of time or milk type is indicated on each panel when *p* <0.05.

The average milk yield per 12 h interval decreased over time (*p* <0 .0001) from 27.3 kg (pre-DEX) to 15.3 kg at 24 h post-DEX, remained low (19.0 kg) at 36 h post-DEX, then returned to baseline values by 60 h post-DEX (Figure 2A). There was a parallel, transient decline in the calculated energy-corrected milk yield (Sjaunja et al., 1991), from 31.3 to 22.6 kg per 12 h interval by 24 h post-DEX (*p* < 0.01). For the one cow that was removed from the study, no foremilk could be collected by machine milking at 24 h post-DEX and required oxytocin for ejection, such that the yield and composition data reported for that cow at 24 h reflect the entire volume collected as hindmilk following oxytocin (Supplemental Data Sheet S1, Figure 2).

The composition of all fore- and hindmilk samples collected from QTR4, from time 0 to 60/72 h post-DEX, is depicted in Figure 2 for the *n* = 3 cows that completed the study. Values for the additional cow excluded from the analysis are presented as Supplemental data (Supplemental Data Sheet S1, Figure 2). The concentration of lactose in milk changed over time (*p* = 0.003), was higher in fore-*versus* hind milk (*p* <0 .0001), and in foremilk was lower at 24 h after DEX compared to 0 h (*p* <0 .05) before returning to baseline levels (Figure 2B). There was no effect of time on the fat content of milk (*p* >0.05, Figure 2C), albeit its concentration in foremilk was increased at 24 h post-DEX compared to 0 h, before returning to baseline (*p* <0.05). As expected, there was a higher fat content in hind milk (*p* = 0.0003).

The concentration of total protein (Figure 2D) and casein (Figure 2E) in milk changed over time (*p* <0.0001) and was increased at 24 h post-DEX (*p* < 0.05), without differences between fore- and hindmilk. The concentration of LALBA in milk changed over time (*p* = 0.005), where in foremilk its concentration was lower at 36 h compared to 72 h after DEX (Figure 2H; *p* = 0.007). Total solids changed over time (*p* = 0.008) reflecting increased levels in foremilk at 24 and 36 h post-DEX (Figure 2F), whereas solids in hindmilk were unchanged. There was no change in the SCC of milk over the experimental period in either fore- or hindmilk (Figure 2G).

**FIGURE 3:**
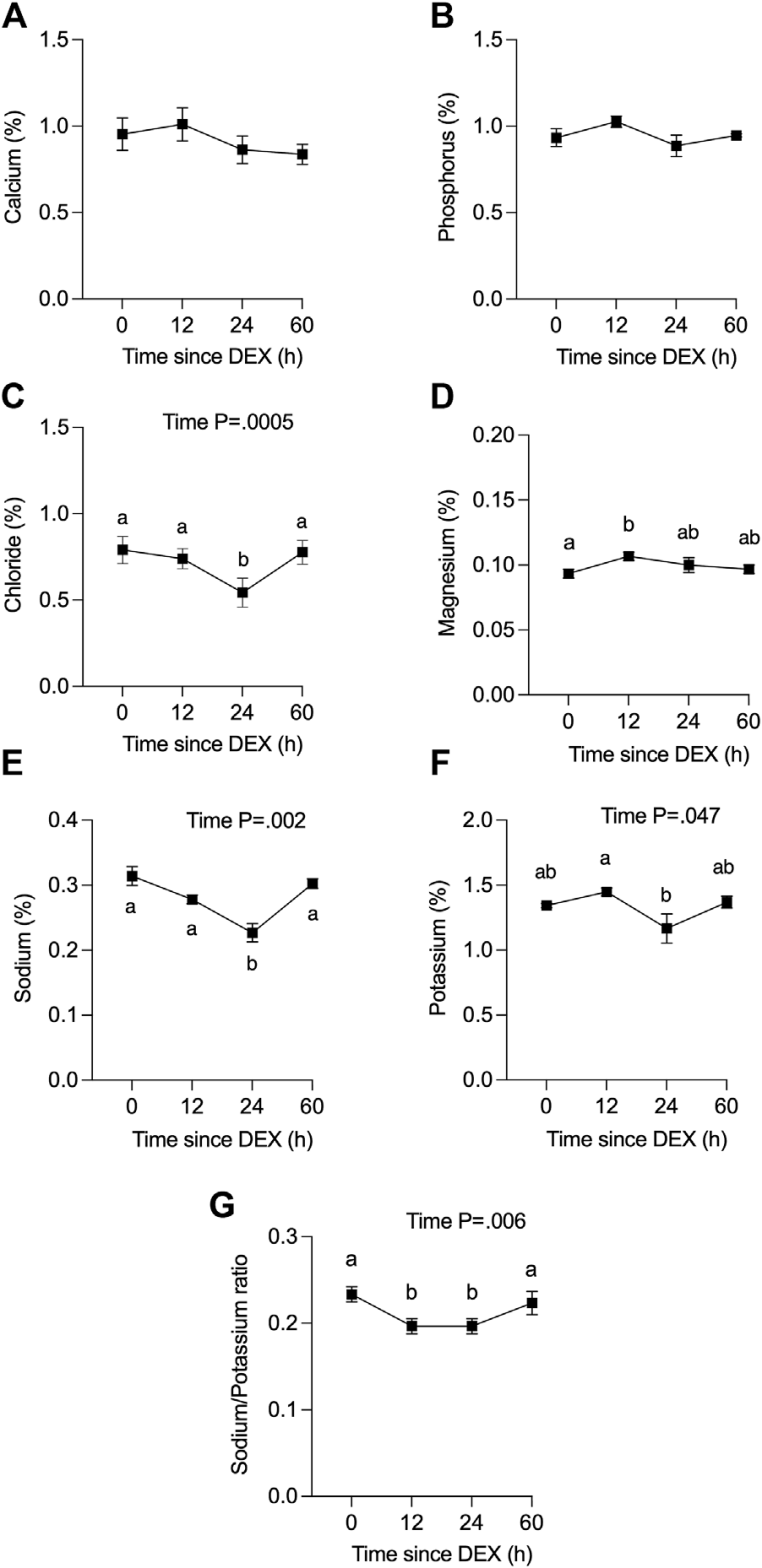
Effect of dexamethasone (DEX) on the concentration (%) of minerals in combined fore- and hindmilk. **(A)** calcium, **(B)** phosphorus, **(C)** chloride, **(D)** magnesium, **(E)** sodium, **(F)** potassium and **(G)** sodium:potassium ratio. Data are means ± SEM (n = 3 cows). Time 0 h (h) is for a single datapoint per cow (not an average of previous values). a, b Means with different superscripts are different (*p* <0.05). The *p*-value for a main effect of time is indicated on each panel when *p* <0.05.

We also determined the level of electrolytes in QTR4 foremilk collected at 0, 12, 24, and 60 h relative to DEX (Figure 3). There was no change in the concentration of Ca or P in response to DEX (Figures 3A,B). The concentration of Cl (Figure 3C, *p* < 0.005) and Na (Figure 3E, *p* < 0.05) was decreased at 24 h in response to DEX compared to all other time points, while Mg was higher at 12 h compared to 0 h (Figure 3D, *p* < 0.05). There was a small reduction in milk K at 24 h compared to 12 h (Figure 3F, *p* < 0.05). The Na/K ratio decreased (Figure 3G) from 0.23 to a nadir of 0.20 at 12 and 24 h, then returned to baseline at 60 h (*p* < 0.05). Data for the omitted cow are presented in Supplementary Figure S3.

### Effect of DEX on the mammary transcriptome

**FIGURE 4:**
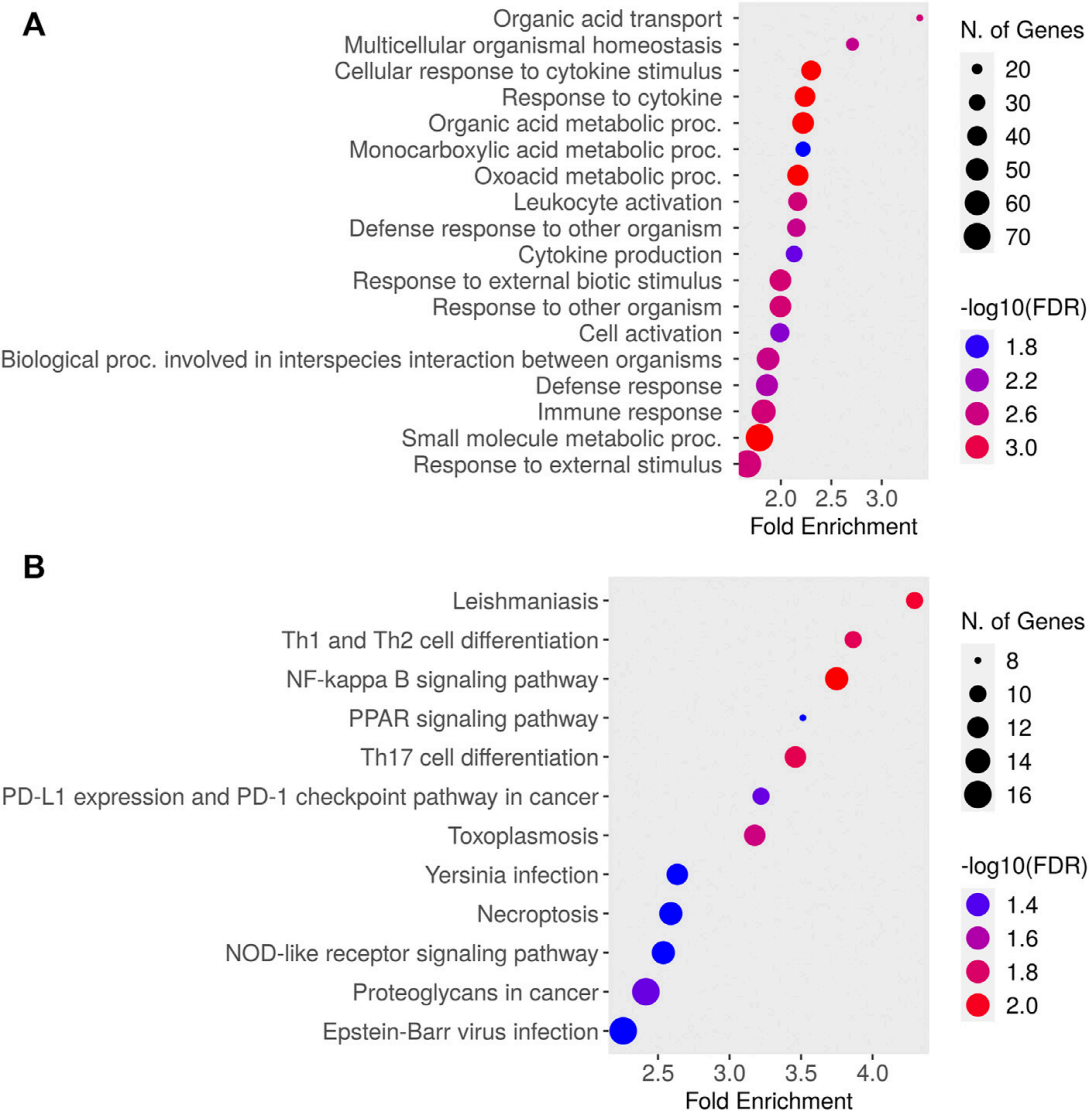
Dotplot of **(A)** Gene Ontology (GO) Biological Processes and **(B)** Kyoto Encyclopedia of Genes and Genomes (KEGG) pathways enriched for genes differentially expressed in the lactating udder at 12 h post dexamethasone.

**FIGURE 5:**
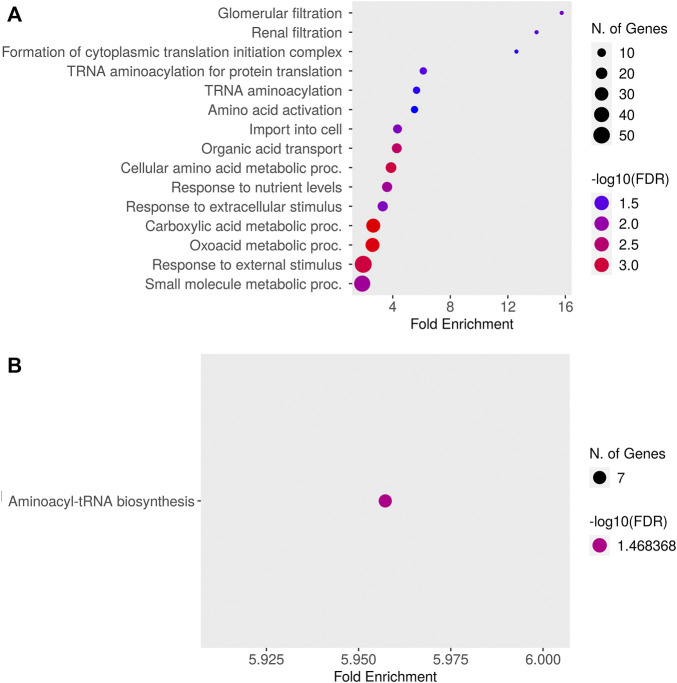
Dotplot of **(A)** Gene Ontology (GO) Biological Processes and **(B)** Kyoto Encyclopedia of Genes and Genomes (KEGG) pathways enriched for genes differentially expressed in the lactating udder at 24 h post dexamethasone.

Relative to baseline gene expression at time 0, the expression of 519 and 320 genes was altered at 12 and 24 h after DEX, respectively (Table 1). Figure 4 shows the GO biological processes and KEGG pathways enriched in the lactating mammary gland at 12 h post-DEX (FDR<0.05), while Figure 5 shows the GO biological processes and KEGG pathways enriched at 24 h post-DEX (FDR<0.05). Of note, by 72 h post-DEX, no genes differed in their expression relative to that at time 0 (Table 1), highlighting that the mammary gland transcriptome was completely restored by 72 h after DEX.

**TABLE 1:** Pairwise comparisons for differential gene expression in response to DEX. *Adjusted *p* <0.05, *n* = 3 cows.

Comparison (hours)	Number of genes*
0 v 12	519
0 v 24	320
0 v 72	0
12 v 24	99
12 v 72	519
24 v 72	516

Regression analysis across the entire study period identified seven genes (RDH12, TUBA1B, AZGP1, CEP57L1, SESN1, EPHX2, and TMEM35B) having an expression profile that associated with the change in milk yield (adjusted *p* <0.05). By contrast, no genes had an expression profile across time that associated with the change in milk fat or lactose content. After adjusting for gene expression changes attributable to altered milk yield, the expression of one gene (ENSBTAG00000047609) retained a negative association with the change in milk fat content over time (adjusted *p* <0.05).

We next defined changes in the expression of individual genes specifically at 12 and 24 h after DEX. After 12 h, 519 genes were differentially expressed compared to time 0. The top ten most significant biological process ontologies (Table 2) were “immune system process”, “actin cytoskeleton reorganization”, “positive regulation of fat cell differentiation”, “negative regulation of protein kinase activity”, “heart contraction”, “cGMP-mediated signaling”, “female gonad development”, “cellular response to cAMP”, “response to bacterium”, and “response to drug”. By 24 h after DEX, the expression of 320 genes had changed, where the top ten biological process ontologies were “translation”, “cytoplasmic translation”, “formation of cytoplasmic translation initiation complex”, “intrinsic apoptotic signaling pathway in response to DNA damage by p53 class mediator”, and “response to oxidative stress”. Of the 204 genes having upregulated expression at 24 h after DEX, 136 were functionally annotated in the DAVID database. Of note among these, the expression of twenty unique genes was significantly upregulated (FDR<0.05) and belonged to three biological process ontologies: translation (RPL34, RPS27, RPS13, RPS2, EEF2, RPL13A, ENSBTAG00000047136, RPL5, MRPL10, RPL23A, RPL23, RPL24, RPL13, SLC25A3, and RPL30), translational initiation (EIF2S3, EIF3E, EIF3D, EIF3H, EIF3F, and EIF1), and formation of translation preinitiation complex (EIF2S3, EIF3E, EIF3D, EIF3H, and EIF3F).

**TABLE 2:** The top ten most significant biological process ontologies for genes that were upregulated or downregulated in response to DEX.

Name	Count	Up	Down
Response to bacterium	8	CAV1, COLEC12, IRAK1, TICAM2, LPO	CFD, MPEG1, TLR4
immune system process	36	TSPAN6, B4GALT1, CAV1, HSP90AB1, CD46, IMPDH2, CNOT7, COLEC12, STAT3, IRAK1, DDIT4, TICAM2, PHB, MPP1, LPO, GCNT1, FST, PTX3, SNX10	VAV1, TLR3, BLA-DQB, TMEM106A, ALOX15, PSMB9, ENPP3, CD320, LGALS9, CASP4, CFD, LGALS1, SOD1, AQP3, MSN, PSMB10, TLR4
actin cytoskeleton reorganization	1	—	PDLIM4
positive regulation of fat cell differentiation	1	—	MEDAG
negative regulation of protein kinase activity	7	CAV1, HMGCR, DNAJA1, GSKIP	FABP4, TRIB2, WARS1
heart contraction	3	SNTA1, CAV1	SOD1
cGMP-mediated signaling	1	PDE2A	—
female gonad development	2	FST	SOD1
cellular response to cAMP	1	FDX1	—
response to drug	4	—	FBP1, SOD1, PDE2A, SLC1A3

### Effect of DEX on expression of candidate genes/pathways within the mammary gland

**FIGURE 6:**
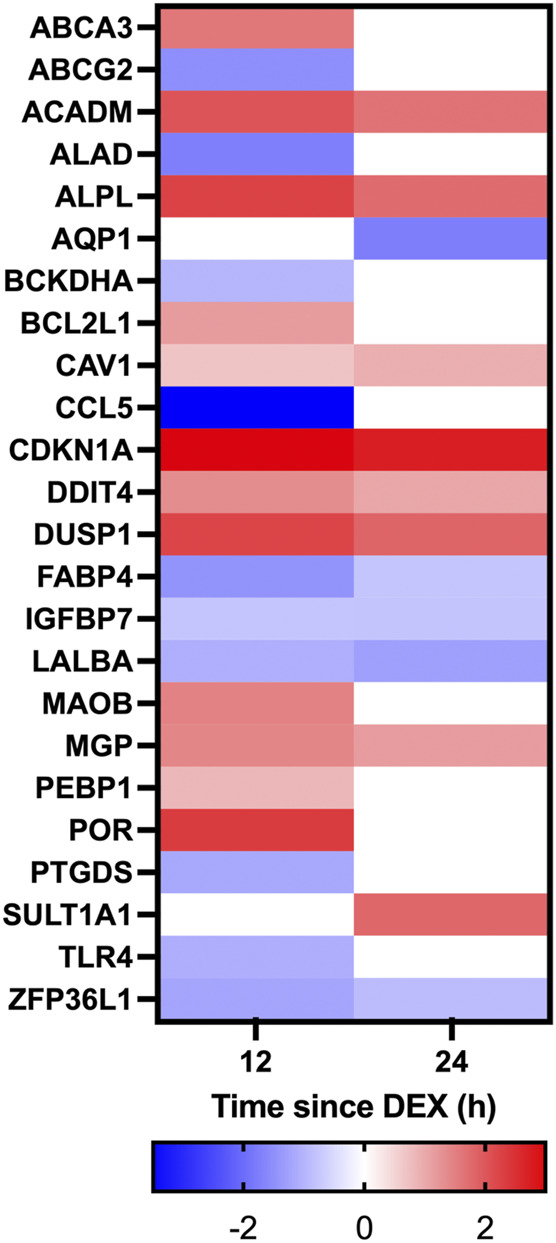
Heatmap depicting changes in differential gene expression in response to dexamethasone (DEX) for genes categorized under the gene ontology term “Response to corticosteroid”. Only genes with a significant (adjusted *p*-value <0.05) change in expression for either 12 v 0 h (h), or 24 v 0 h, and a log fold change between −2 and 2 in response to DEX are shown. Gene expression data is for *n* = 3 cows.

**FIGURE 7:**
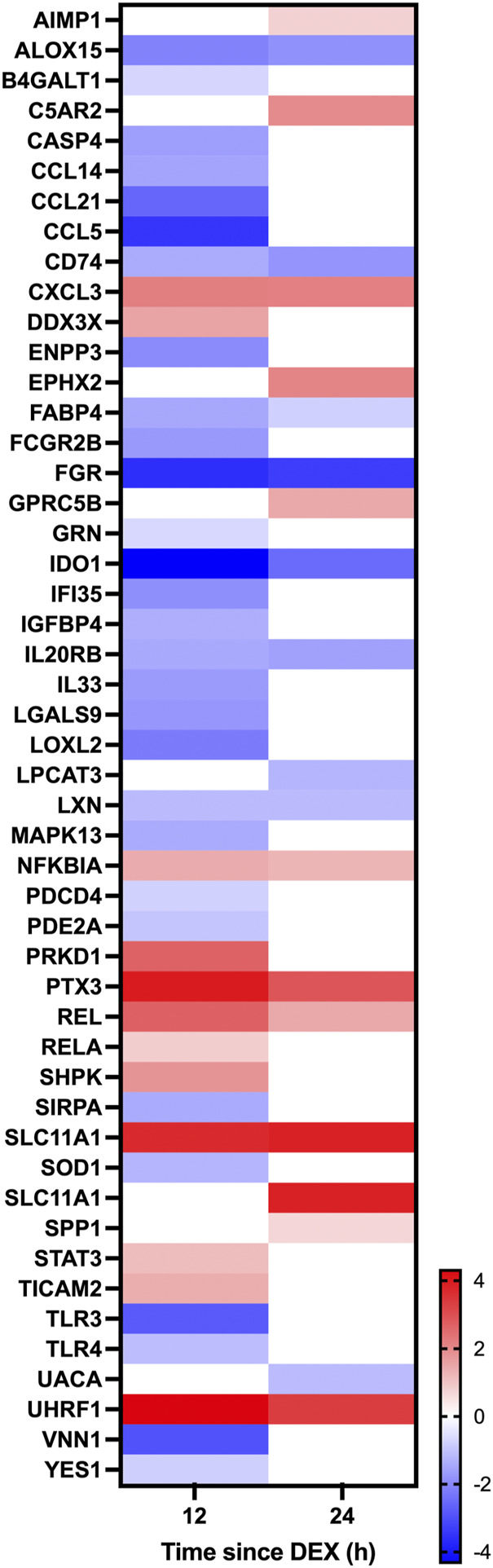
Heatmap depicting changes in differential gene expression in response to dexamethasone (DEX) for genes categorized under the gene ontology term “Inflammation.” Only genes having a significant (adjusted *p*-value < .05) change in expression for either 12 v 0 h (h) or 24 v 0 h, and a log fold change between −4 and 4 in response to DEX are shown. Gene expression data is for *n* = 3 cows.

Given the established global effects of GC on gene expression (Ratman et al., 2013), we further examined the effect of DEX on candidate GC targets in the mammary glands including the local inflammasome (Rhen and Cidlowski, 2005), blood flow (Kerachian et al., 2009; Ozmen et al., 2017), and the integrity of tight junctions between the mammary epithelium (Stelwagen et al., 1998; Stelwagen et al., 1999). Within the category “response to corticosteroid” there was 10 and 5 genes having expression that was downregulated at 12 and 24 h, respectively, while 12 and 8 genes had upregulated expression at 12 and 24 h, respectively (Figure 6). Within the category “inflammation” there was 29 and 9 genes downregulated at 12 and 24 h after DEX, respectively, and 12 and 11 genes having expression that was upregulated at 12 and 24 h (Figure 7). Data for the omitted cow are presented in Supplementary Figure S4. As noted previously, there were no signs of mastitis for any of the cows during the experimental period.

Several genes categorized under “blood vessel diameter maintenance” were differentially regulated in response to DEX, including three that were downregulated (ADD3, FGG, and SOD1) and 5 that were upregulated (CAV1, CBS, HMGCR, KCNMB4, KCNMB4, and SNTA1) at 12 h post-DEX, of which three genes (CAV1, KCNMB4, and SNTA1) remained upregulated at 24 h. Among genes defined by the GO term “tight junction”, two were downregulated (CLDN15, ESAM) and 3 were upregulated (USP53, C1QTNF5, and YBX3) at 12 h post-DEX, while only two genes (DLG3 and YBX3) were upregulated by 24 h post-DEX, where the expression of YBX3 was upregulated at both time points.

### Effect of DEX on genes in the lactose synthesis pathway

**FIGURE 8:**
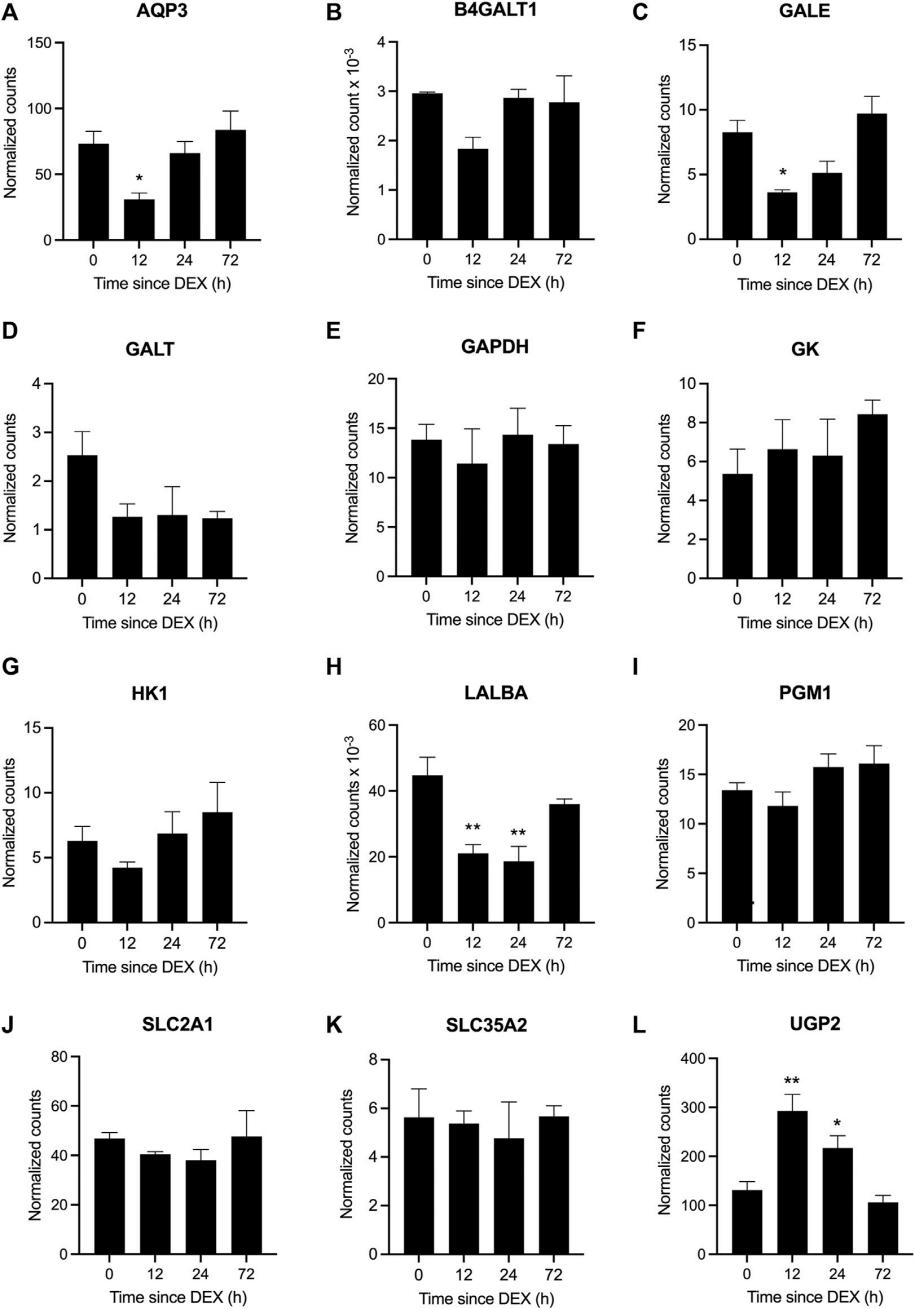
Differential gene expression in response to dexamethasone (DEX) for genes in the lactose synthesis pathway. Data represent mean ± SEM (n = 3 cows) for the genes **(A)** aquaporin 3, AQP3, **(B)** beta-1,4-galactosyltransferase 1, B4GALT1, **(C)** UDP-galactose-4-epimerase, GALE, **(D)** galactose-1-phosphate uridylyltransferase, GALT, **(E)** glyceraldehyde-3-phosphate dehydrogenase, GAPDH, **(F)** glycerol kinase, GK, **(G)** hexokinase 1, HK, **(H)** lactalbumin alpha, LALBA, **(I)** phosphoglucomutase 1, PGM1, **(J)** solute carrier family 2 member 1, SLC2A1, **(K)** solute carrier family 35 member A2, SLC35A2, and **(L)** UDP-glucose pyrophosphorylase 2, UGP2. *P <0.05 and **P <0.01 versus time = 0 hours (h).

A cohort of genes involved in the lactose synthesis pathway was among those that were differentially expressed at 12 and 24 h post-DEX (Figure 8). Specifically, *AQP3, GALE,* and *LALBA* were all downregulated at 12 h (adjusted *p* <0.05; Figures 8A,C,H), while the expression of *UGP2* was upregulated at 12 h (adjusted *p* <0 .05). The expression of *LALBA* continued to be suppressed at 24 h post-DEX, while *UGP2* expression remained elevated (adjusted *p* <0.05; Figure 8H,L). The expression of other genes associated with the lactose synthesis pathway including *B4GALT1, GAPDH, GALT, GK, HK1, PGM1, SLC2A1,* and *SLC35A2* was unaffected by DEX, where some genes showed numerical changes in expression that may have been statistically underpowered due to omission of the fourth cow (Figure 8B,D–G,I–K, and Supplemental Data Sheet S1, Supplementary Figure S5). Of note, the expression of another major milk protein, *CSN2* was unchanged in response to DEX (data not shown).

### Predicted upstream regulators

Not surprisingly, several GC were among the top upstream regulators identified using the Drug Signatures Database in Enrichr, where flumetasone, diflorasone and fluorometholone were the top 3 predicted upstream regulators for the lists of differentially-expressed genes at 12 and 24 h post-DEX. Indolo [3,2-b]carbazole, mastinib (AB1010), and etynodiol HL60 were the top three predicted upstream regulators for genes having downregulated expression at 12 h, while valrubicin, fenbendazole, and ofloxacin were the predicted upstream regulators for genes with downregulated expression at 24 h.

## Discussion

Here we investigated the effect of elevated systemic GC, as occurs during various states of stress, on milk synthesis and gene expression by the mammary glands of dairy cows. Consistent with earlier reports (Hartmann and Kronfeld, 1973; Shamay et al., 2000b), a single, high dose of DEX administered to multiparous cows transiently suppressed the yield and lactose content of milk, and increased its protein content, without negatively affecting its concentration of fat. Lactose synthesis returned to baseline levels by 36 h post-DEX, after a nadir at 24 h, alongside the transitional normalization of milk yield, composition, and the expression of *LALBA* and *B4GALT1*, concomitant with the restoration of euglycemia. Our transcriptomic analysis highlighted that DEX downregulated several aspects of lactose synthesis and function in the mammary epithelium, spanning from precursor uptake and hexose metabolism through to lactose synthesis, in a time-dependent manner. This suppression of lactose synthesis is in keeping with its recognized and critical role as the major osmole in milk (Sadovnikova et al., 2021a). These changes were also accompanied by a marked reduction in water secretion, where water transporters in the mammary glands localize to the capillary endothelium (AQP1) or mammary epithelium (AQP3), although limited data exist for how their expression is regulated in the mammary gland (Mobasheri and Barrett-Jolley, 2014).

Central to the reduction in lactose output was the downregulation of gene expression for several components of the lactose synthase complex (LSC) within 12 h post-DEX, including for the essential modifier protein, LALBA, which remained suppressed out to 24 h. At the same time, expression of *B4GALT1*, the enzymatic component of the LSC, was suppressed at 12 h, but was then restored by 24 h. By contrast, gene expression for another major milk protein, *CSN2*, was unchanged following DEX exposure. This acute and specific suppression of LSC activity in response to elevated GC aligns with the differential regulation of milk protein gene expression across a range of GC concentrations in rodent mammary tissue *ex vivo/in vitro* (Sadovnikova et al., 2021b). Specifically, the expression of LALBA *ex vivo/in vitro* is stimulated by low, relatively-physiological concentrations of GC, whereas high concentrations are suppressive (Ono and Oka, 1980; Nagamatsu and Oka, 1983). By contrast, the positive effect of GC on gene expression for milk proteins such as CSN2 and whey acidic protein was distinctly sigmoidal and monotonic across a range of GC concentrations (Ono and Oka, 1980; Nagamatsu and Oka, 1983). We should highlight that these reductions in *LALBA* and *B4GALT1* expression and overall lactose synthesis coincided with evidence for other coordinated changes implicated in precursor transport and hexose metabolism (Sadovnikova et al., 2021a), despite not always reaching statistical significance due to the challenged sample size within this study. For example, there were indications for reduced abundance of *GLUT1* (*SLC2A1*) at both 12 and 24 h in keeping with its established role during glucose uptake (Zhao, 2014). Likewise, the downregulated expression of *AQP3* not only aligns with its role as a water transporter but also for transporting glycerol (Hara-Chikuma and Verkman, 2006), which may have led to a reduction in its availability as an alternative precursor for galactose synthesis (Sadovnikova et al., 2021a). Similarly, a non-significant decline in *HK1* expression at 12 h post-DEX coincided with the anticipated reduction in glucose uptake and demand, where hexokinase activity controls 80% of glucose being metabolized for lactose synthesis (Xiao and Cant, 2005). At the same time, transient downregulation of *GALE* at 12 (and non-significantly at 24 h) would have reduced the accumulation of UDP-galactose being provided for lactose synthesis, thereby accumulating glucose-1-phosphate. The increased and sustained expression of *UGP2* at 12 and 24 h post-DEX would have then potentially rerouted this excess glucose-1-phosphate to UDP-glucose for alternative metabolism, such as toward glycogen synthesis. Indeed, Emerman et al. (1980) proposed that glycogen accumulation was an important shunt during lactogenesis in the absence of maximal lactose synthesis, and that stored glycogen could be recycled for lactose synthesis during its subsequent activation around parturition. Combined, these data point to a mechanism whereby elevated GC activates the rapid and targeted downregulation of lactose synthesis in the mammary glands across multiple steps, beginning at the level of gene expression, thereby affording a reversible and rapid glucose-sparing benefit to the female as would be physiologically-warranted during an acute, stressful event. This mechanism of glucose diversion away from the mammary glands to realize the transient suppression of milk yield and lactose output, alongside increased/stable fat and protein content, was similarly evident during insulin-induced hypoglycemia (Rook et al., 1965). Indeed, diverting glucose away from the mammary glands results in the rapid cessation of milk synthesis, as occurred in perfused udders (Hardwick et al., 1963). These examples substantiate how the glucose-sparing effect of acute DEX, as documented by others (Kronfeld and Hartmann, 1973; Shamay et al., 2000b), underlies its therapeutic benefit when administered to ketotic dairy cows (Gordon et al., 2013).

The complete reversal of milk production, composition and the mammary transcriptome after DEX highlights the plasticity of the mammary epithelium and lactose synthesis in response to elevated GC. These changes mirror those recorded during several examples of the transient reversal of milk production loss following exposure to a range of stressors including elevated temperature (Collier et al., 2017; Becker et al., 2020), conversion to once-daily milking (Littlejohn et al., 2010), and after the systemic response to mastitis (Shuster et al., 1991). On this last front, our data contribute toward an understanding of the negative systemic regulation of milk synthesis during mastitis. As reviewed by Shangraw and Mcfadden. (2022), an informative model for addressing this question has been the local challenge of one mammary quarter with lipopolysaccharide (LPS) to induce transient hypogalactia in adjacent glands, alongside altered milk composition, reduced lactose output, hyperglycemia and increased circulating GC. In reviewing the mechanisms involved as well as their own data, Shangraw and Mcfadden. (2022) suggested that either inflammatory cytokines derived from the LPS-treated gland, or circulating GC that are elevated in response to either intramammary or intravenous LPS exposure, are the likely mediators underlying this hypogalactia. A comparison of the differentially-expressed gene sets among our data with those of Shangraw et al. (2021) revealed several notable similarities. Of the 14 genes that were differentially expressed (upregulated) in the untreated glands at 3 h after adjacent intramammary LPS (Shangraw et al., 2021), 5 were also upregulated at 12 h post-DEX (*ARRDC2, RGS1, CDKN1A, NFKBIA, and PTX3*; adj *p* <0 .05), where all these genes have been described as sharing anti-inflammatory properties. Shangraw et al. (2021) also identified that *AQP1* expression was downregulated during LPS-induced hypogalactia, where we recorded that *AQP1* was downregulated at 12 h (albeit adj *p* = 0.11), and moreso at 24 h (adj *p* = 0.014), in keeping with a likelihood that its expression changed after the reduction in lactose synthesis. Along similar lines, Littlejohn et al. (2010) found that transcriptomic changes within the udder following its transition to once-daily milking, alongside a reduction in milk yield, LALBA and lactose synthesis, mirrored several of those we recorded following DEX. Of note, in both our study and that of Littlejohn et al. (2010) there was increased expression of *RELA* and downregulation of the toll-like receptors (*TLR2* during once-daily milking, and *TLR3* and *TLR4* after DEX). The fact that these types of immune-associated changes occurred in the mammary glands across all 3 studies [(Shangraw et al., 2021) and our present data], absent any pathogenic response, points to GC-induced activation of local mediators as being a likely mechanism at play during various stress responses. In turn, the systemic increase in GC during states such as mastitis would serve to acutely suppress glucose uptake by all quarters, thereby prioritizing the availability of glucose for the immune system over that for milk production. Certainly the glucose requirements of the immune system are significant, where that of a lactating cow consumes >1 kg during an acute LPS challenge (Kvidera et al., 2017). While there is wide acceptance that the glucose demands in support of a normal lactation are directed by homeorhetic adaptation (Bauman and Currie, 1980), these transcriptomic profiles support the notion that the glucose requirements of an activated immune system trump those of the mammary glands in order to maintain homeostasis (Bradford and Swartz, 2020). We posit that systemic GC levels serve as the central mediator of this balance.

## Conclusion

Our data show that DEX administered to lactating dairy cows leads to the temporary and specific suppression of milk yield and lactose synthesis due to reduced expression of *LALBA* and other lactose synthesis intermediates within the mammary glands. We conclude this response allows the homeorrhetic repartitioning of glucose toward immune activation during physiological stress responses. This work is an important step towards understanding how stress and exogenous GC contribute to transient hypogalactia.

## Data Availability

The datasets presented in this study can be found in online repositories. The names of the repository/repositories and accession number(s) can be found below: https://www.ncbi.nlm.nih.gov/geo/query/acc.cgi?acc=GSE217369.
