# Supplementary figures and images for: Transcriptomic changes underlying glucocorticoid-induced suppression of milk production by dairy cows
Source: Front Genet. 2022 Dec 6;13:1072853. doi: 10.3389/fgene.2022.1072853 (PMC9763454; doi:10.3389/fgene.2022.1072853)

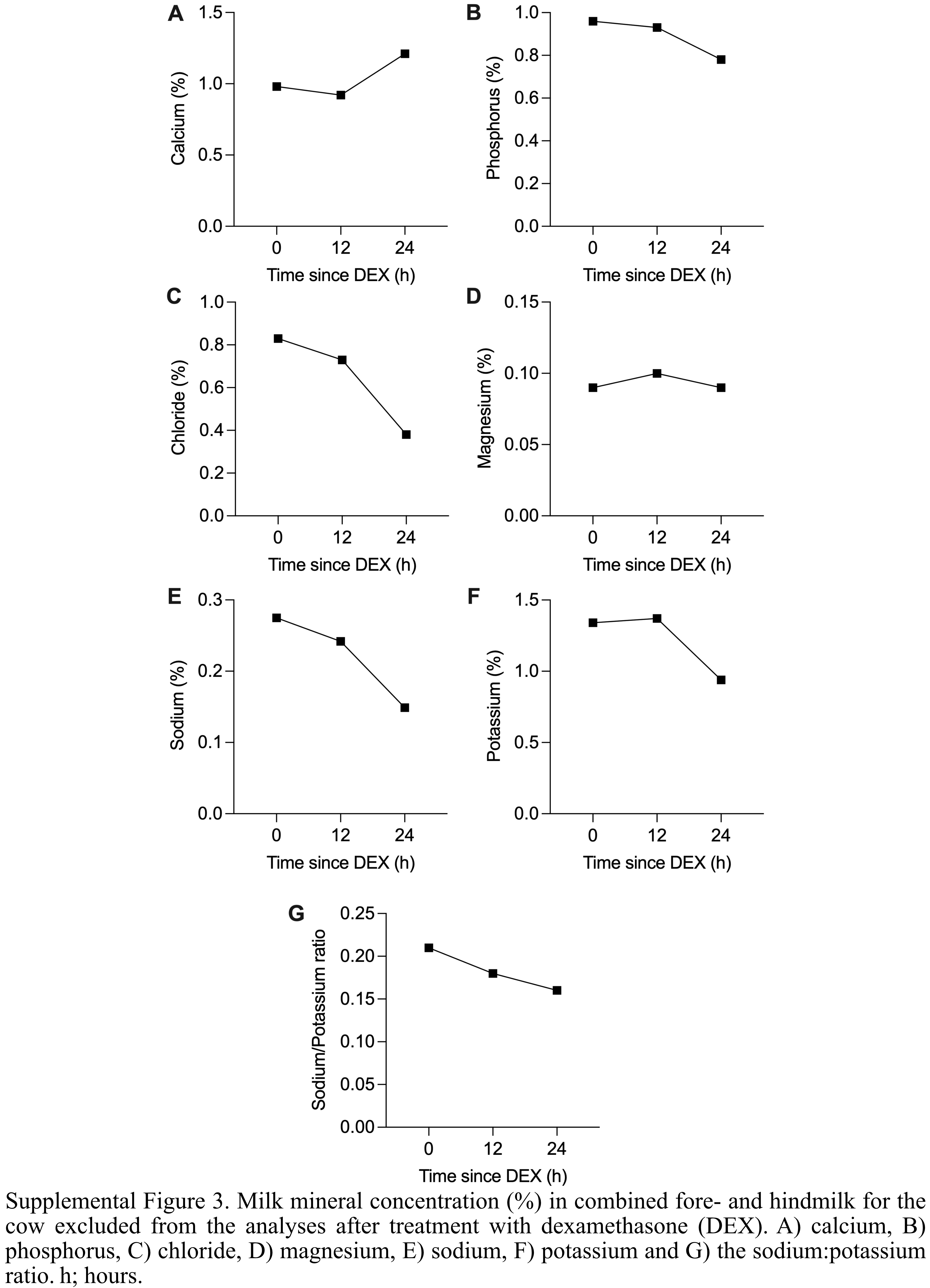

Supplement: Supplementary file 1 [file Image3.TIFF]

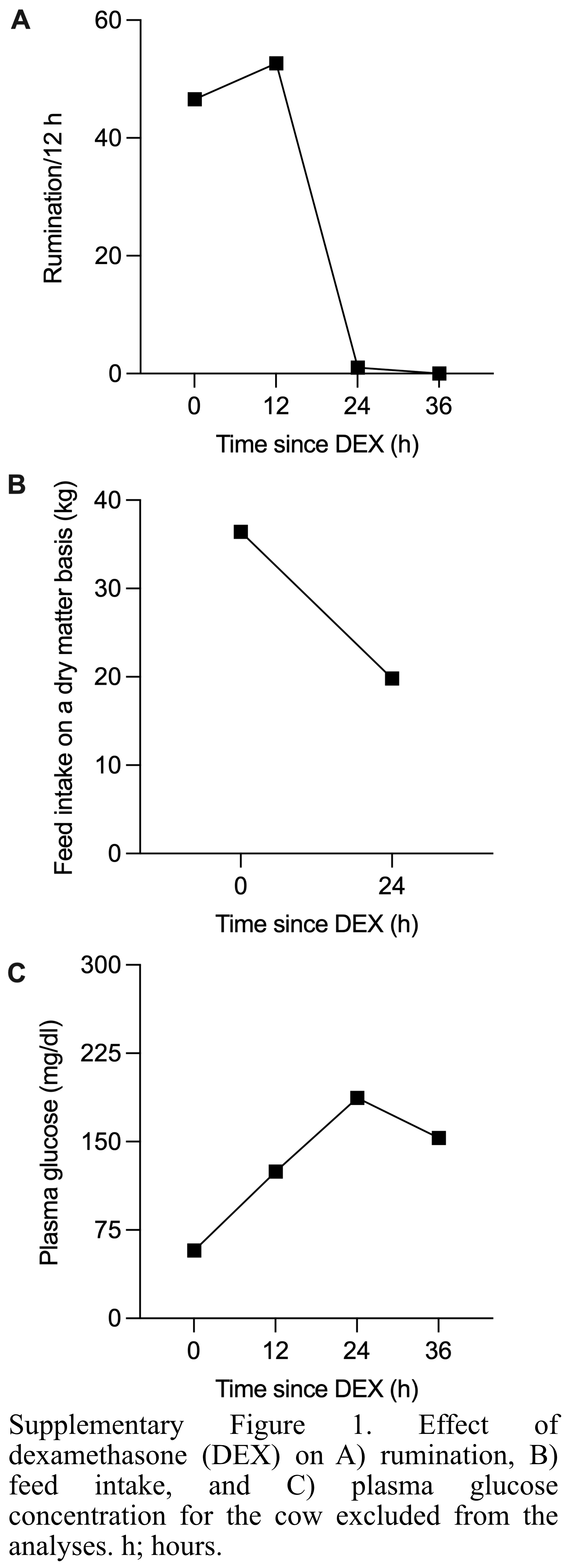

Supplement: Supplementary file 2 [file Image1.TIFF]

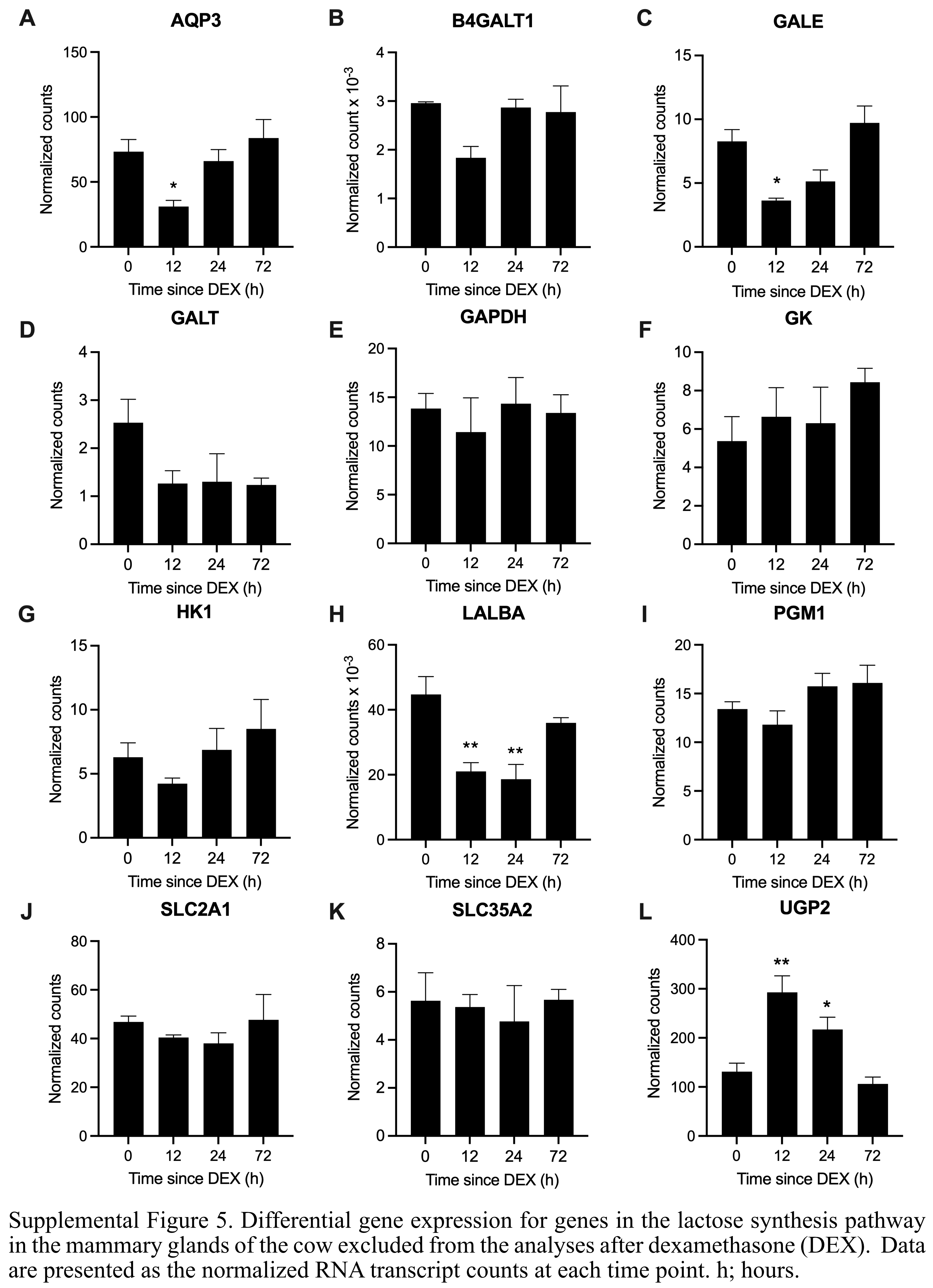

Supplement: Supplementary file 4 [file Image5.tiff]

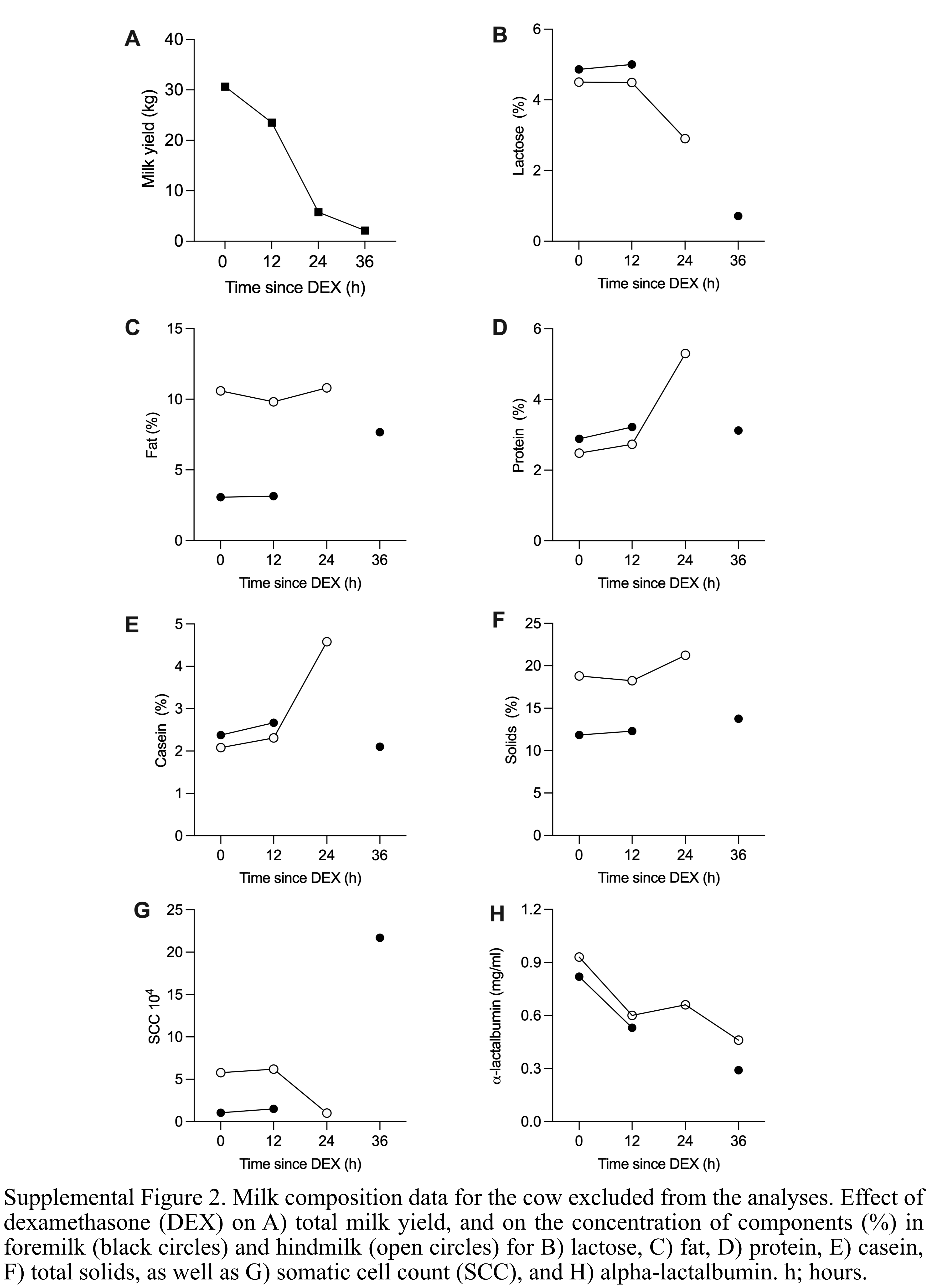

Supplement: Supplementary file 5 [file Image2.tiff]

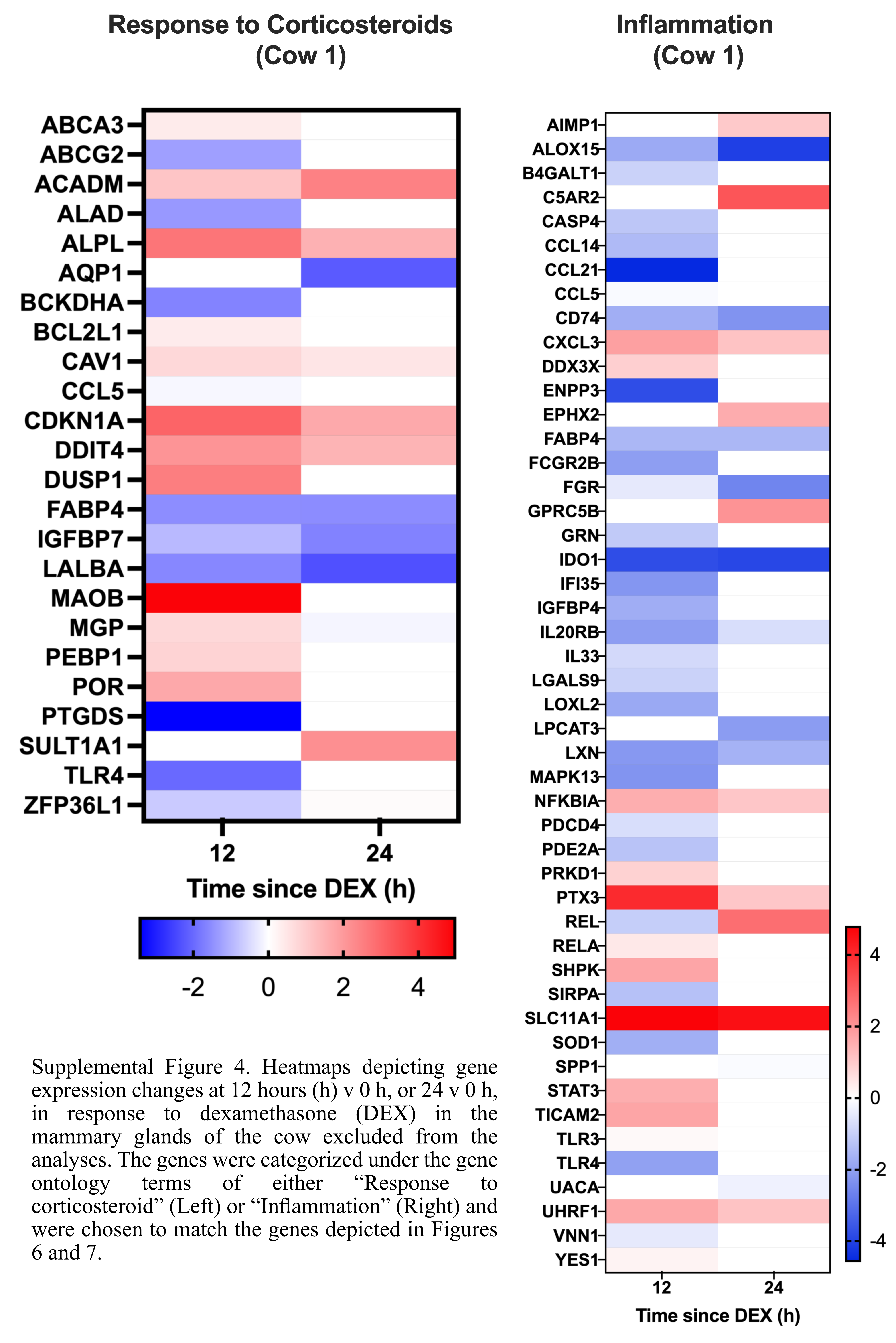

Supplement: Supplementary file 6 [file Image4.tiff]
