# Supplementary material for: Transcriptomic changes underlying glucocorticoid-induced suppression of milk production by dairy cows
Source: Front Genet. 2022 Dec 6;13:1072853. doi: 10.3389/fgene.2022.1072853 (PMC9763454; doi:10.3389/fgene.2022.1072853)
Supplement: Supplementary file 3 [file Table1.DOCX]

Supplementary Table

**Supplementary Table 1.** Nutrient composition of the total mixed ration (dry matter basis)

| Crude protein (%) | 19.1 |
| --- | --- |
| Soluble protein (% of crude protein) | 26 |
| Acid detergent fiber (%) | 19.4 |
| Neutral detergent fiber (%) | 31.5 |
| Lignin (%) | 5.95 |
| Non-NDF carbohydrate (%) | 35.4 |
| Starch (%) | 19.5 |
| Crude fat (%) | 5 |
| Ash (%) | 9.0 |
| Total digestible nutrients (%) | 68.5 |
|  |  |
| Net energy, lactation (Mcal/kg) | 1.61 |
